# Ingestion accidentelle de pile bouton en intra-œsophagien

**DOI:** 10.11604/pamj.2016.23.1.8442

**Published:** 2016-01-05

**Authors:** Djafar Mamoudou, Mounia Idrissi

**Affiliations:** 1 Service de Pédiatrie, CHU Hassan II, Fès, Maroc; 2 Département de Médecine et Spécialités Médicales, FSS, Université Abdou Moumouni de Niamey, Niger

**Keywords:** Corps étranger, pile bouton, endoscopie, Ingestion, foreign bodies, upper endoscopy

## Image en médecine

Les corps étrangers digestifs chez l'enfant représentent un motif fréquent de consultation. La fibroscopie œso-gastro-duodénale permet de confirmer le diagnostic d'ingestion de corps étranger mais permet aussi son extraction dans la majorité des cas. Un enfant de 2 ans sans antécédents pathologiques notablesa été admis aux urgences pédiatriques pour notion d'ingestion accidentelle de corps étranger remontant à trois jours. L'enfant était asymptomatique depuis l'ingestion. L'examen physique à l'admission était sans particularité. Une radiographie du thorax a été réalisée qui avait mis en évidence un corps étranger radio-opaque enclavé au niveau cervical. De face il s'agissait d'une opacité ronde à double contour régulier; de profil d'une opacité linéaire qui confirmé la position postérieure du corps étranger œsophagien par rapport aux clartés antérieures du larynx, de la trachée et de la carène. Cette opacité est caractéristique de l'image de pile bouton enclavé au niveau du 1/3 supérieur de l’œsophage dont l'extraction immédiate s'impose au risque des complications (ulcérations, perforation digestives). Le diagnostic différentiel se pose avec l'ingestion d'une pièce de monnaie qui se traduit par une opacité aussi ronde mais avec un seul contour et dont l'extraction peut être différer en l'absence des signes cliniques. L'endoscopie digestive haute a été réalisée qui avait mis en évidence une pile bouton intra-œsophagienne et l'extraction a été faite à l'aide d'une pince tripode sans incidents.

**Figure 1:**
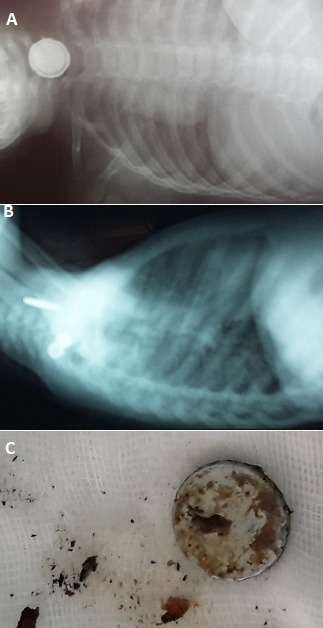
A) radiographie thoracique de face montrant l'opacité ronde cervicale; B) radiographie thoracique de profil montrant l'opacité linéaire postérieure par rapport aux clartés de la trachée; C) pile bouton après extraction

